# Influence of long-term exposure to simulated acid rain on development, reproduction and acaricide susceptibility of the carmine spider mite, Tetranychus cinnabarinus

**DOI:** 10.1673/2006_06_19.1

**Published:** 2006-09-21

**Authors:** Jin-Jun Wang, Jian-Ping Zhang, Lin He, Zhi-Mo Zhao

**Affiliations:** 1 Key Laboratory of Entomology and Pest Control Engineering, College of Plant Protection, Southwest University, Chongqing 400716, P. R. China

**Keywords:** Tetranychus cinnabarinus, acid rain, development, reproduction, susceptibility

## Abstract

Development, reproduction and acaricide susceptibility of Tetranychus cinnabarinus (Boisduvals) (Acari: Tetranychidae) were investigated after long-term (about 40 generations) exposure to various levels of acid rain; pH 2.5, 3.0, 4.0, and 5.6. Deionized water (pH 6.8) served as a control. The mites were reared on eggplant leaves at 28°C, 80%RH and a photoperiod of 14:10 (L:D) in the laboratory. The results showed that the duration of the immature stage was significantly affected by acid rain exposure. The shortest duration (8.90 days) was recorded for populations exposed to pH 5.6 acid rain, while the longest duration (9.37 days) occurred after exposure to pH 2.5 acid rain. Compared with the control population, adult longevity was shortened with an increase in acidity. Similarly, the oviposition duration was also shortened by an increase in acidity. Statistically, female fecundity did not differ significantly between pH 5.6, pH 4.0 and control populations, but did differ significantly between the control population and those exposed to pH 2.5 and pH 3.0 acid rain. This suggested that the mite suffered reproductive defects after long-term exposure to acid rain with higher acidity (pH 2.5 and 3.0). The intrinsic rate of increase among different populations was not significantly affected, but the net reproductive rate of populations exposed to pH 2.5 and 3.0 acid rain was significantly less than pH4.0, 5.6, and control populations. Bioassay results showed that after long-term exposure to acid rain, susceptibility of the mites to two acaricides, dichlorvos and fenpropathrin, did not change significantly.

## Introduction

The threat of acid rain to the global biosphere has become an environmental problem of worldwide importance. Increased acidity of many lakes and soils, alteration of the bio-communities’ equilibria, and decline of forests have been shown to be related to acid rain (Shaefer 1992; Driscoll et al. 2001; Menz and Seip 2004). The impact of acid deposition, usually in the form of simulated acidic rain, on vegetation, including the direct (primary) and indirect (secondary) effects through the influence on soils, has been well documented (Cowling 1982; Lee 1982; Linthurst et al. 1982; Treshow 1984; Shriner 1986; Treshow and Anderson 1989; Dai et al. 1998; Menz and Seip 2004). Acid deposition also influences host-plant susceptibility and suitability to insect herbivores (Bedford 1986; Riemer and Whittaker 1989; Heliövaara and Väisänen 1993; Zhang et al. 2005). While there are numerous studies on the effects of air pollution on insects, the majority of these investigations were conducted in forest systems under the influence of particulate and/or gaseous pollutants. Most references to insect population dynamics state that air pollution is one of the predisposing factors that influences the susceptibility of forest stands to outbreaks (Grodzki et al. 2004). A number of studies have been done concerning effects of air pollution on phytophagous insects in agricultural systems (Zhang et al. 2004).

Regions that have been most affected by acidic deposition include Europe, eastern North America, and Southeast Asia, especially central and southern China (Kuylenstierna et al. 2001). Sulphur emissions have played the dominant role in acidic deposition in these regions. Sulphur emissions in China decreased in the late 1990s, increased from 1999 to 2000, and remained stable up to 2002 (Li and Gao 2002). Chongqing is a city in China that is seriously polluted by acid rain. The average frequency of acid precipitation in Chongqing was about 70–90% and the lowest pH value of acid precipitation was 3.6. Acid rain in Chongqing has caused soil acidification, reduced forested areas and crop yield, and intensified insect pest infestation (Zhao et al. 1999). Based on our investigation, the infestation of the carmine spider mite, Tetranychus cinnabarinus (Boisduvals), a serious mite pest of vegetable and fibre crops, is positively correlated with the frequency of acid precipitation. Our previous work has focused upon determining the biological and physiological effects of acid rain on the development and reproduction of T. cinnabarinus (Zhao et al. 1999; Wang et al. 2004; Zhang et al. 2004). The results indicated that there was no acute lethality of sprayed simulated acid rain of pH 4.0–3.0 on this mite. However, the development and reproduction of the mite was affected by the direct application of acid rain. Mite population dynamics and host injury increased significantly after acid rain (pH 4.0) was applied to both on the mites and the host plant. Biochemical analysis showed that the peroxidase, superoxide dismutase, catalase and acid phosphatase might be relevant to population changes of mites under acid rain pressure.

Demographic toxicology incorporating life table parameters is applied in meaningful comparative studies. Life table parameters for populations unexposed or exposed to various concentrations of a toxicant or pollutant can be incorporated, and ensuing population responses can be compared. This approach combines ecological and toxicological parameters, resulting in better predictions about the effects of toxicants at the population level. Ultimately, the power of the intrinsic rate of increase (*r_m_*) as an ecologically meaningful bioassay parameter for toxicology studies lies in the fact that this statistic is based on both survivorship (*l_x_*) and fecundity (*m_x_*). Estimates of *r_m_* are environmentally specific and must be viewed in the context of the experimental conditions used (Stark et al. 2003). The relative fitness of a population can be evaluated by the average population size when the population has achieved an equilibrium by natural selection after several generations in a given environment (Carson 1961). To date, the study on the long-term impacts of acid rain on herbivorous insect and mite populations (i.e. impact on insect and mite fitness over several generations) is lacking. The present study was undertaken to evaluate the effects of long-term exposure to acid rain on the development, reproduction and pesticide susceptibility of T. cinnabarinus.

## Materials and Methods

### Mites

The stock culture of carmine spider mite, T. cinnabarinus (Boisduval), was originally collected from the cowpea bean (Vigna sinensis Endlicher) in Chongqing, China. This culture was maintained on potted kidney bean (Phaseolus vulgaris L.) in a insect rearing room at 28 ± 1°C, 75–80%RH, and a photoperiod of 14:10 (L:D). This colony was maintained for more than two years before acid rain treatment. Voucher specimens were deposited in the insect collection of the Southwest University, Chongqing, P. R. China.

### Simulated acid rain treatment

An initial solution was created by adding to deionized water the mean final concentration of 5.35mg/L (NH_4_)_2_SO_4_, 0.36 mg/L Na_2_SO_4_, 0.97 mg/L MgSO_4_, 6.80 mg/L CaSO_4_, 1.52 mg/L KNO_3_, 0.51 mg/L NaNO_3_, 0.77 mg/L MgCl_2_, and 0.29 mg/L NaF as determined from data obtained by the Tongyuan Monitoring Station, Chongqing for the ionic composition of rain (Wang and Qin 1989). An amount of H_2_SO_4_ was added to bring the initial solution to pH 5.6, 4.0, 3.0 or 2.5. Deionized water, pH6.8, was used as a control. The pH values of all solutions were checked with a pHS-4C meter (Chengdu Instrument Inc., Chengdu, China).

### Host plant

Eggplants (Solanum melongena L.) grown in pots (7.5 cm height, 4.5 cm diameter) were used for mite rearing. Plants were replaced every 30 days throughout the study. The plants were fertilized with a controlled Hoagland nutritional solution (Hoagland and Arnon 1950) and watered with deionized water as required. The experiment was conducted in the insect rearing room described above. For experimental use the mites were transferred from the stock colony to egg plants. In all experiments, simulated acid rain of different pH values were applied to the potted eggplant with mites from a portable overhead sprinkler system at a height of 75 cm from the ground level. The plants were sprayed every two days with 150 ml of simulated rain per plant each time. The mite colony was transferred to new plants every 30 days or shorter throughout the study. Similar mite population densities were maintained for all acid rain treatments. After one year and three months (about 40 generations), the mites from each treatment were used for developmental, reproductive and acaricide susceptibility studies.

### Development and reproduction of mite

This experiment was conducted to test the effects of long-term exposure to acid rain with different acidicity on the development and reproduction of the mite. In this experiment, leaves of eggplants sprayed with deionized water were used, and all leaves used were from the same phyllotaxis. Petri dishes (12 x 1.5 cm), each with a piece of water-soaked foam plastic inside, were used as observation units. The water-soaked foam plastic was covered with a layer of Whatman No. 1 filter paper. Five female and two male adult mites from each treatment were transferred to a detached eggplant leaf placed on the filter paper. The mites were held at 28ºC for a 4 hour oviposition period, after which the adults were removed. Petri dishes were placed in growth chambers (Jiangnan Instrument Inc., China) at 28ºC, 80%RH, and a photoperiod of 14:10 (L:D). Eggs were checked twice daily and after hatching, 20 larval mites were transferred individually using a camel’s-hair brush to a small area isolated by strips of absorbent cotton on the lower surface of a detached leaf. Individual mites were checked twice daily for ecdysis (i.e., for life stage and developmental period) and survivorship. The exuviae were counted to determine molting. Mites were transferred to new leaves every 2 or 3 days throughout the study.

After eclosion, male and female adults were paired. Each pair was transferred daily to a fresh leaf disk and eggs laid were counted under a stereomicroscope. Observations were recorded daily until the last female died.

### Bioassay

This experiment was carried out to test the relationship between the susceptibility of the mites to acid rain and acaricides. The susceptibility of the mites after exposure to acid rain treatment to two acaricides, dichlorvos and fenpropathrin, purchased from Chongqing Pesticide-Chemical Company, P. R. China, was assessed following the recommended methods of the FAO (Dennehy et al. 1983). Various concentrations of each insecticide were tested until a satisfactory range was ascertained. Six concentrations were used in the final analysis. Each population was bioassayed by the slide-dip method using six treatments of acaricides, and a control. The acaricides were diluted with water to the required concentrations, and a slide with about 30 adult female spider mites, 3 –5 days old, was dipped into the solution for 5s then left to dry. Each treatment was replicated three times. The slides were left in an incubator with temperature and humidity controlled at 25°C and 75% relative humidity. Mortality was assessed after 24 h. Mites that did not move after stimulation from a camel’s hair brush were scored as dead.

### Data analysis

The life history parameters of mites on eggplant leaves were analyzed among different populations using the general linear model (GLM) procedure of SAS (SAS Institute, 1988) and means were separated by least significant difference (LSD) test. Life table of different strain statistics were calculated as described byHulting et al. (1990). The differences in *r_m_* values among populations were also analyzed using Newman-Keul sequential tests (Sokal and Rohlf, 1981) based on jackknife estimates of variance for *r_m_* s (Meyer et al., 1986). For any difference between 2 *r_m_*s from the sequence, in which the *r_m_*s were arrayed in order of magnitude, to be significant at the α level, it must be equal to or greater than:


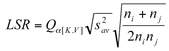


where *K* is the number of *r_m_*s in the set whose range is tested, and V is the degrees of freedom. The *n_i_* and *n_j_* were sample sizes of the 2 *r_m_*s, and *Q_α[K.V]_* is a value from the table of the studentized range.*S_av_*^2^ is the weighted average variance of *r_m_* and it is calculated as follows:


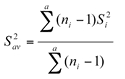


where *a* equals the number of *r_m_s* to be tested, the sample size of the*i*th *r_m_* is *n_i_*. *S_i_*^2^ is the jackknife estimate of the variance for the *i*th *r_m_*.

The bioassay results were corrected for control mortality using Abbott’s (1925) formula. According to the results of bioassay, the log-concentration against probit-mortality lines was developed, and the comparison of mortalities between the populations was made by the concentrations required for 50% mortality (LC_50_).

## Results

### Immature development of T. cinnabarinus

After long-term exposure to acid rain treatment (about 40 generation), the developmental periods of immature stages of T. cinnabarinus varied significantly among populations exposed to different acid rain conditions (Table 1). For egg development, the longest duration was recorded for pH 4.0 acid rain exposure population (4.83 days), while the shortest was for pH 2.5 acid rain exposure populations (*F* = 31.472; df = 4, 595; *P* < 0.0001). In contrast, the length of the larval stage in mites exposed to pH 5.6 and pH 4.0 treatments were both less than for the pH 6.8 control population, while the population exposed to pH 2.5 acid rain was longer than control population (*F* = 17.822; df = 4, 549; *P* < 0.0001). The length of the nymphal stage in mites exposed to the pH 2.5 treatment was significantly longer than those of the other four populations (*F* = 10.490; df = 4, 439; *P* < 0.0001). The long-term exposure to acid rain also affected the pre-oviposition period significantly (*F* = 1.853; df = 4, 214; *P* < 0.0001). The total length of immature stages of mites exposed to the pH 6.8, 5.6, 4.0, 3.0, and 2.5 treatments were 9.09, 8.9, 9.25, 8.92, and 9.37 days, respectively.

**Table 1:**
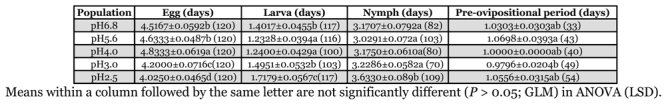
Developmental periods (days, M±SE) of immature stages of different T. cinnabarinus populations after exposure to acid rain for 40 generations

### Reproduction and life table parameters of T. cinnabarinus

Mean female longevity was significant different among the five populations (*F* =13.729; df = 4, 214; *P* < 0.0001), and was longest (12.70 days) for the control population and shortest (8.83 days) for the population exposed to pH 2.5 acid rain (Table 2). More than 90% of the females began to lay eggs within 24 h after copulation and they continued laying eggs for their entire adult life under all acid rain treatments. However, the numbers of eggs laid per female and oviposition durations were all significantly affected by the different pH acid rain exposure history. In general, the mites exposed to acid rain laid fewer eggs and had shorter oviposition periods than those of control populations. The mean sex ratio for all populations was strongly biased toward females (81.66%). The oviposition duration of pH2.5 acid rain exposure population was shorter than that of other populations (*F* = 14.745; df = 4, 214; *P* < 0.0001).

**Table 2:**
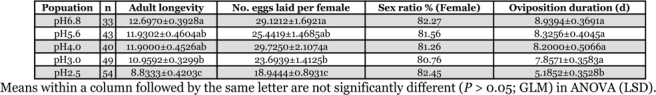
Fecundity and longevity (M±SE) of different T. cinnabarinus populations after exposure to acid rain for 40 generations

The intrinsic rate of increase (*r_m_*), net reproductive rate (*R_0_*), mean generation time (*MT*), and population doubling time (*t*) and finite rate of increase (λ) were calculated for populations of T. cinnabarinus exposed to different pH acid rain (Table 3). The *r_m_* values of different populations were not significantly different *(P* > 0.05). There were significant differences in *R_0_* values between mites exposed to pH 2.5 and 3.0 and the control population (*P* < 0.05), however, *R_0_* values of the other three populations were not significantly different from each other or from the control population (*P* > 0.05). The finite rate of increase (λ) of different populations was ~1.16–1.17. The mean generation time of pH 4.0 was longest (14.24 days) and that of pH 2.5 was shortest (12.31 days). Population doubling time of different populations ranged from 4.48 to 4.66 days. The relative fitness (*R_f_*, which is defined as the ratio of *r_m_* values between acid rain exposed and control populations) of pH 5.6, pH 4.0 and pH 3.0 were 1.013, 1.033 and 1.07 respectively, while that of pH 2.5 was 0.993. In general, the relative fitness of different populations did not change significantly.

**Table 3:**
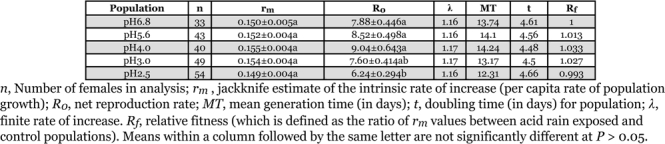
Comparison of life table parameters of different T. cinnabarinus population after exposure to acid rain for 40 generations

### Dichlorvos and fenpropathrin susceptibility of T. cinnabarinus

The susceptibility of T. cinnabarinus to dichlorvos and fenpropathrin are shown in Table 4. The slope of the probit lines showed that the populations were relatively homogeneous with regard to susceptibility. This suggested that after 40 generations of exposure to acid rain, the susceptibility of T. cinnabarinus to dichlorvos and fenpropathrin did not change significantly.

**Table 4:**
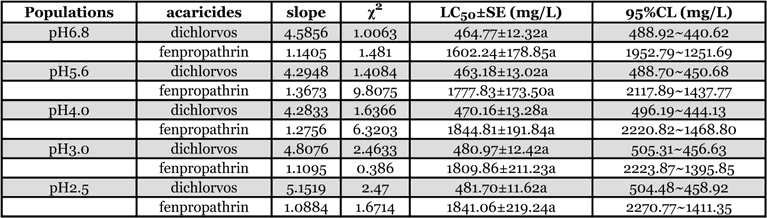
Susceptibility of different T. cinnabarinus populations to two acaricides after exposure to acid rain for 40 generations

## Discussion

The demographic toxicological approach is not only a measure of the total toxic effect, but also sub-lethal effects that are not perceptible can be evaluated. Results of several studies have indicated that sub-lethal effects can be very subtle and affect populations at concentrations lower than the traditional concentration response curve (Stark et al. 2003). Studying the effects of acidic water on populations of Daphnia pulex, Walton et al. 1982 found that 96-hour toxicity tests detected no effects of acidic water, but negative effects were easily discerned using an evaluation of the intrinsic rate of increase. Other studies reported that acid rain enhanced growth and development of insects (Palokangas et al. 1995; Redak et al. 1995; Stinner et al. 1988). Although there was no acute lethality of sprayed simulated acid rain on T. cinnabarinus, the development and reproduction of the mite was restrained by direct application of acid rain (pH 4.0-3.0) (Zhao et al. 1999). On the other hand, Wang et al. 2004 reported that development and reproduction of T. cinnabarinus were enhanced through the host plant-mediated impact of simulated acid rain (pH 5.6-3.0). The T. cinnabarinus population and host plant injury increased significantly after acid rain (pH 4.0) was applied to the mites and the host-plants (Wang and Zhao 2002). Furthermore, Zhang et al. 2004 reported that after long-term exposure to acid rain, physiological aspects of the mite, including some enzymes, were enhanced significantly. Such physiological changes might be related to development, reproduction and acaricide susceptibility of the mites.

The present study clearly demonstrates that immature development and adult reproduction was affected significantly after long exposure to acid rain. The mean generation times at pH 5.6 and pH 4.0 populations were longer, but those at pH 3.0 and pH 2.5 populations were shorter than control. However, doubling times for populations exposed to pH 5.6, pH 4.0 or pH 3.0 acid rain were shorter than controls. In addition, compared with the control population, the relative fitness of mite populations exposed to pH 5.6, 4.0, and 3.0 acid rain were all greater than 1.0. Moreover, Zhang et al. 2005 reported that weak acid rain (pH > 4.0) promoted the activities of three protective enzymes (SOD, PID and CAT) of eggplant leaf and its growth. The growth of T. cinnabarinus was also promoted because the content of soluble sugar phosphate and soluble protein changed in eggplant leaves making them more favorable to feeding by mites. However, strong acid rain (pH<3.0) inhibited the growth of both host plant and the mite. This might be the reason for mite infestations in agricultural areas prone to high rates of acid deposition.

The intrinsic rate of increase (*r_m_*), is often used to determine an insect’s ability to thrive on various hosts (Kerns et al. 1989). Comparison of *r_m_* values often provides considerable insight beyond that available from independent analyses of several life history parameters (Petitt et al. 1994). Following Carson (1961), the relative fitness of a population can be evaluated by the average population size when the population has achieved an equilibrium by natural selection after several generations in a given environment. Our previous study showed that the development and reproduction of mites varied significantly when acid rain was sprayed on mites or host-plants; the jackknife estimate of *r_m_* of pH4.0~3.5 was significantly higher than that of the control population (Zhao et al. 1999; Wang et al. 2004). However, after long-term exposure to acid rain, despite the effects on the development and reproduction of mites, jackknife estimates of*r_m_* and *R_f_* were similar when the simulated acid rain was stopped. This implies that these changes were temporal and able to recover. Acid rain did not induce genetic changes in the mites and only induced life parameter changes that were plant-mediated (Zhang et al. 2005).

Zhang et al. 2004 reported that after long-term exposure to acid rain, the activity of several enzymes closely related to pesticide resistance including peroxidase, superoxide dismutase, catalase, phosphatase and carboxylesterase varied significantly among populations of mites exposed to different acidity treatments. However, bioassay studies reported here indicate that the susceptibility of different T. cinnabarinus populations to dichlorvos and fenpropathrin did not vary. Thus, although acid rain did influence development and reproduction of T. cinnabarinus by direct or indirect effects, acid rain did not affect susceptibility of T. cinnabarinus to acaricides.

The present study together with our previous studies give further evidence that T. cinnabarinus is remarkably tolerant to a wide range of acidification conditions, and that acid rain, particular pH4.0-5.6, will definitely increase the infestation of the mite. As acid rain significantly affects areas of China, including areas of agricultural and natural biotic importance, further detailed studies investigating the impact of acid rain upon T. cinnabarinus-host plant interactions are warranted.
